# Veterinary Medical Association of New Jersey

**Published:** 1886-01

**Authors:** Wm. Herbert Lowe

**Affiliations:** Secretary


### VETERINARY MEDICAL ASSOCIATION OF NEW JERSEY.

The sixth regular meeting of this association was held at the West Jersey Hotel, in Camden, Thursday, December 10, 1885.

The President, Dr. Wm. B. E. Miller, in the chair. The following members were present: Drs T. B. Rogers, of Westville; H. W. Rowland, of Jersey City; J. W. Hawk, of Newark; C. K. Dyer, of Mount Holly; W. P. Smith, of Trenton; L. R. Sattler, of Newark; J. C. Dustan, of Morristown; W. P. Humphrey, of Elizabeth; M. A. Pierce, and Wm. Herbert Lowe, of Paterson.

Among those present as visitors, were Drs. T. C. Sanford, of Asbury Park; F. W. Hilyard, of Mount Holly; W. H. Williams, of Fellowship, and Wm. H. Iszard, M.D., of Camden.

Dr. Smith proposed Dr. L. P. Hurley, of Camden, for membership, and Dr. Lowe proposed Dr. E. R. Mercer, of Montclair. The names of both gentlemen were referred to the Board of Censors, who reported favorably, and they were duly elected. Dr. Dyer, proposed Drs. T. C. Sanford, of Asbury Park and F. W. Hilyard, of Mount Holly. The Board of Censors did not report in regard to the two last named candidates. Action to be taken at the next regular meeting.

Two of the honorary members, Dr. E. M. Hunt, Secretary of the State Board of Health, and Dr. Julius Gerth, State Veterinarian of Nebraska, sent letters regretting their inability to attend. A letter of regret was also received from Dr. W. H. Pendry, Secretary of the N. Y. State Veterinary Society.

The Treasurer's report was presented and accepted.

The new Constitution and By-Laws, prepared by Dr. Lowe since the incorporation of the Association under the Act of the Legislature for the advancement of Veterinary Science and Art, were presented by him. Their consideration occupied most of the first session, but they were finally adopted substantially as prepared. That portion entitled *The Code of Ethics*, caused much discussion, but was adopted to read as follows:—

"It shall be regarded as a grave offence for any member to speak disrespectfully of another, or by insinuation or otherwise to injure his reputation or professional standing. It behoves each member to do as he would wish to be done by, and to cultivate that sense of honor and propriety which should invariably distinguish the professional man. As the Association aims to protect the privileges and immunities of its members, it is expected that they will exercise their abilities in extending and enriching the domain of comparative medicine and surgery, and in advancing the interests of the profession."

"No veterinary physician should prescribe for a patient having been treated by another member of the profession unless the former has relinquished the case, or that the owner announces that he has dispensed with his services."

"In case of consultation, the consulting veterinarian should, so far as he can do so conscientiously, sustain the surgeon in charge of the case, and in no way either by word or act, promote his own interests at the expense of his brother practitioner. He whose practice is based on an exclusive dogma, or who rejects the accumulated experience of the profession, or ignores the aids of anatomy, physiology, pathology and organic chemistry, shall not be considered a fit associate in consultation.

"While it is essential for the veterinarian consulted to ascertain the true nature of the case, he should carefully withhold all discussion of the subject till his brother practitioner and himself meet in private for deliberation. When a conclusion is reached, it will be the duty of the attending veterinarian to state the results to his client, in presence of the consulting veterinary surgeon. No opinions should be delivered which are not the result of previous deliberations and concurrence.

"When diversity of opinion exists, it may be proper to refer the case to several veterinarians, in good standing, or a court-medical. Still, in most cases, mutual concessions should render this unnecessary.

"All discussions in consultation should be confidential.

"Qualifications, and not intrigue or artifice, should constitute the foundation for successful practice."

"Any advertisement or announcement, beyond the name and address, shall be deemed unprofessional.

"Any advertisement or announcement of nostrums, secret remedies, panaceas, and all things of a like nature, shall be deemed unprofessional.

"The promising of radical cures, and the procuring of certificates regarding the same, or of operations with a view to advertisement would be deemed reprehensible. It is also reprehensible for veterinarians to give certificates relating to patent medicines, or promoting their use in any way.

"There is no profession whose members are more tempted to practice double dealing, than the veterinary, as for instance, in the giving of opinions regarding the purchase and sale of horses. Any member guilty of that offence shall be expelled from the Association.

"Any proposed alteration or amendment of the Constitution and By-Laws shall be submitted in writing to the Board of Trustees, and should their approval be given, action will be taken at the next regular meeting of the Association. A two-thirds vote will be necessary to confirm the same."

Prior to the second session the members of the Association visited the farm of Michael Feenfer, a milkman at Pavonia, near Camden. The object of the visit was to ascertain the sanitary conditions of the farm. Certain publications had been made by a human practitioner, Dr. C. R. Earley, of Ridgway, Elk Co., Pa., taking Drs. Miller and Dyer to task for an alleged want of professional knowledge, and as to their performance, or rather what he regards as the non-performance of certain duties in representing the State Board of Health. After making examinations the Association sustained the views and actions of Drs. Miller and Dyer. A committee consisting of Drs. Hawk, Smith and Pierce, were appointed to draft resolutions. They presented the following, which were adopted and signed by all of the members present:—

"We the special committee appointed by the Veterinary Medical Association of New Jersey, in session here this day, to draft suitable resolutions condemning the grossly outrageous article published in the *Sanitarian*, written by C. L. Earley, a physician residing at Ridgway, Elk Co., Pa., in which he attacks unjustly Drs. W. B. E. Miller and C. K. Dyer, do present the following:—

"*Resolved*, That we, as a veterinary association, do condem the article published as a malicious falsehood on these gentlemen, whose ability and standing in the profession is well known to all of us, and we endorse the actions of Drs. Miller and Dyer in their treatment of the herd at Mr. Feenfer's, at Pavonia.

"*Resolved*, That the Association having in a body visited the herd of cattle and premises of Mr. Feenfer, testify that they found them to be in a first class condition ; that the sanitary and ventilating arrangements were good.

"*Resolved*, That a copy of these resolutions be published in the Camden papers, and that one be sent to the *Sanitarian*."

Dr. Rogers, of Westville, read a paper vindicating Drs. Miller and Dyer.

Dr. Lowe paid a tribute to the late Dr. Weeks, of Paterson, before introducing the following resolutions, which were passed and ordered entered upon the minutes

"*Whereas*, the late Dr. Arthur P. Weeks was a worthy member of our Association. therefore be it

"*Resolved*, That we express our sincere sorrow for the loss of so true a friend of veterinary science ; and that we unite in deep sympathy with his relatives in their affliction.

"*Resolved*, That the death of our friend at the early age of 27 years, and especially as his young life gave promise of a highly useful and honorable career, we feel all the more deeply the present bereavement.

"*Resolved*, That these expressions of our sorrow and sympathy be recorded in the minutes of our Association, and that a copy of the same be forwarded to his family."

Quite a number of interesting cases were related, and views interchanged on the same.

Dr. Dyer moved that the Secretary be appointed a committee of one, with power, to take charge of such printing as may be necessary, including the new Constitution and By-Laws, Stationery and Certificates of Membership. It was so ordered.

The Association adjourned to meet in Morristown, the second Thursday of next April.

WM. HERBERT LOWE, D.V.S.,
*Secretary.*

